# *FLT3 *mutation incidence and timing of origin in a population case series of pediatric leukemia

**DOI:** 10.1186/1471-2407-10-513

**Published:** 2010-09-27

**Authors:** Patrick Chang, Michelle Kang, Anny Xiao, Jeffrey Chang, James Feusner, Patricia Buffler, Joseph Wiemels

**Affiliations:** 1 Department of Epidemiology and Biostatistics, UCSF, Helen Diller Cancer Research Building, 1450 3rd Street, San Francisco, CA 94158, USA; 2 Children's Hospital and Research Center, Oakland, 747 52nd Street, Oakland, CA 94609, USA; 3 Children's Center for Cancer and Blood Disorders of Northern Virginia, 6565 Arlington Blvd, Suite 200, Falls Church VA 22042, USA; 4 School of Public Health, UC Berkeley, 1995 University Avenue, Suite 460, Berkeley, CA 94704, USA

## Abstract

**Background:**

Mutations in *FLT3 *result in activated tyrosine kinase activity, cell growth stimulation, and a poor prognosis among various subtypes of leukemia. The causes and timing of the mutations are not currently known. We evaluated the prevalence and timing of origin of *FLT3 *mutations in a population series of childhood leukemia patients from Northern California.

**Methods:**

We screened and sequenced *FLT3 *mutations (point mutations and internal tandem duplications, ITDs) among 517 childhood leukemia patients, and assessed whether these mutations occurred before or after birth using sensitive "backtracking" methods.

**Results:**

We determined a mutation prevalence of 9 of 73 acute myeloid leukemias (AMLs, 12%) and 9 of 441 acute lymphocytic leukemias (ALLs, 2%). Among AMLs, *FLT3 *mutations were more common in older patients, and among ALLs, *FLT3 *mutations were more common in patients with high hyperdiploidy (3.7%) than those without this cytogenetic feature (1.4%). Five *FLT3 *ITDs, one deletion mutation, and 3 point mutations were assessed for their presence in neonatal Guthrie spots using sensitive real-time PCR techniques, and no patients were found to harbor *FLT3 *mutations at birth.

**Conclusions:**

*FLT3 *mutations were not common in our population-based patient series in California, and patients who harbor *FLT3 *mutations most likely acquire them after they are born.

## Background

Certain, but not all, chromosome translocations in childhood leukemia are known to be present at birth. This phenomenon of prenatal origin was initially presumed from twin studies where it was observed that mono-amniotic twins always harbored the same translocations (reviewed in [1]). In addition, several studies have shown that specific mutations found at diagnosis in children with leukemia were present at birth, that is, "backtracked" to neonatal Guthrie Cards (blood spots used for newborn screens) (reviewed in [2]). The *MLL *rearrangement in infant ALL "backtracks" in nearly all cases and *TEL-AML1 *is found on 75% of Guthrie cards matched to leukemia cases with the translocation [3-5]. The *E2A-PBX1 *fusion generated by the t(1;19) translocation is a likely exception, with a postnatal origin [6], along with possibly others. These results collectively support a "two hit" model of leukemia, with one hit early in life or *in utero*, and another at a later date in temporal proximity to leukemia diagnosis.

The *FLT3 *gene is located on chromosome 13q12 and encodes a Type III membrane receptor kinase that regulates normal hematopoiesis. Mutations in *FLT3 *in AML occur in approximately 5-15% in children and 25-35% in adults, and account for the most common single gene defect in AML (reviewed in [7]). Several studies have indicated that children and adults with AML and the *FLT3 *mutation have a very poor prognosis. *FLT3 *mutations have also been documented in adult and pediatric ALL. An initial report demonstrated a 14% frequency of *FLT3 *mutations among childhood ALL overall, with mutations concentrated among the cytogenetic subgroups high hyperdiploidy (> 50 chromosomes in diagnostic karyotype) and *MLL*-translocation [8]; more recent studies have indicated a lower overall frequency in childhood ALL (in the 1-8% range) while consistently demonstrating a higher incidence among those with *MLL *rearrangement and high hyperdiploidy [9-13]. The absolute incidence of *FLT3 *mutations in pediatric leukemia is of interest in part because of the existence of several promising FLT3 inhibitors currently under development (reviewed in [14]) such inhibitors are more effective in the presence of FLT3 activation.

Internal Tandem Duplications (ITDs) and activation loop mutations are two unique *FLT3 *mutations that have been characterized. ITDs, which occur on exon 14, are insertions of repeated base pairs that range from 3-400 base pairs each. Activating loop mutations occur on exon 20; they are most commonly missense point mutations that occur at codon 835/836. Point mutations at codon 840, 841 and 842 have been described as well as insertion of base pairs between codons 841 and 842. In adult AML, ITDs comprise the majority of mutations, while in pediatric AML, ITD mutations are less common. It has been proposed that *FLT3 *mutation may be a late event in leukemia, given that FLT3 mutation status are often changed between paired diagnosis and relapse of adult and childhood patients with AML [15-17] or ALL [18]. The present study was conducted to formally assess this timing. We screened a large population case series of pediatric leukemia for *FLT3 *mutations of both types and determined whether *FLT3 *ITD mutations were present at birth by examining DNA on the corresponding Guthrie cards.

## Methods

The research presented here was reviewed and approved by the UCSF Committee on Human Research, protocol # H10806-17300-12, and all study personnel completed appropriate human subjects training courses. Research material was derived from the Northern California Childhood Leukemia Study (NCCLS), and epidemiology study based at UC Berkeley.

### Patients

The patient population consisted of 517 consecutive leukemia patients enrolled in the 9 hospitals participating in the NCCLS during the years 1995 to 2002. Intensive cytogenetic, morphologic, and flow cytometry review [19], parental interviews [20,21] and biologic and environmental sampling [22] were performed, as well as characterization of *NRAS *and *KRAS *mutations [23]. A detailed description of this approximately population-based study design can be found elsewhere [24]. Parental demographic characteristics were provided by the case mother (97.5%) or father (2.5%) through in-person interviews in the home of the parents. A full cytopathological review to distinguish immunophenotype and cytogenetic characteristics was instituted as previously described [19,23]. We employed fluorescence *in situ *hybridization (FISH) to further assess status of two major but cryptic cytogenetic subtypes of childhood ALL, *TEL-AML1 *translocation and high hyperdiploidy, using combined gene-loci specific FISH probes for chromosomes 12 (*TEL*) and 21 (*AML1*), as well as centromere probe for chromosome X. Because > 96% of high hyperdiploid patients have extra copies of *both *chromosomes 21 and X in the same cell [25,26], cases with both extra X and 21 were classified as high hyperdiploidy in the current study. For comparisons with healthy children, controls were individually matched by birthdate, gender, race and ethnicity to cases, and utilized the same questionnaire.

### Mutation Screening

Each sample was amplified by PCR at the two most common sites for the *FLT3 *mutation using Optimase DNA polymerase (Transgenomics) with standard methods. The following primers were used to amplify exon 14-15 where the ITD mutation occurs and exon 20 where missense point mutations commonly occur: ITD-F TATCTGCAGAACTGCCTATTCC, and ITD-R CTTTCAGCATTTTGACGGCAACC; MUT-F CTCCTACTGAAGTTGAGTGTAG, and MUT-R CAGTGAGTGCAGTTGTTTACCA, respectively. Each PCR product was analyzed on a 3% metaphor agarose gel to confirm the presence or absence of the predicted amplicon (large ITDs are indicated by an extra band of larger than expected size). Samples that exhibited ITD mutation (for exon 14 samples) on agarose gel were subsequently sequenced. RFLP analysis was used to screen for missense *FLT3 *mutations (exon 20). *EcoR*V digests wild type *FLT3 *DNA, but does not digest a point mutation at the most common mutation site. Twenty-five μl of the PCR product was digested with 10 U of *EcoR*V for 2 hours at 37°C, followed by incubation at 80°C for 20 minutes to inactivate the enzyme. Digestion products were visualized by electrophoresis on a 3% agarose gel. Positive samples were gel extracted with the Qiagen QIAquick Gel Extraction Kit and confirmed with sequencing. All PCR products were also analyzed on denaturing high pressure liquid chromatography (DHPLC) using a Varian Wave machine with a Transgenomics column at 57.5C; and suspect mutations indicated by aberrant wave patterns were sequenced. This permitted the discovery of mutations outside of codon 835.

### Backtracking

ITD-junction primers that would bind the mutant and would not bind wild type DNA were specifically designed for each mutant. Patient- and mutant-specific primers were designed for each *FLT3 *ITD mutation. The specificity of each mutant specific primer was tested by performing PCR amplifying diluted mutant DNA from a patient in limited dilutions (1:10) within wild type DNA (from peripheral blood cells); mutant specific primers will not bind wild type DNA with optimized assays. This dilution series was used to assess the sensitivity of the assay each time the assay was performed. SYBR green PCR was performed using the mutant specific primer pair designed for each patient to amplify DNA on the diagnostic DNA dilution series and the corresponding Guthrie card for each patient sample where a *FLT3 *ITD mutation was identified.

For point mutation backtracking, we used methods modified from our previous experiments with *KRAS2 *mutation backtracking [27]. Dilution series of patient DNA diluted into wild-type DNA (from peripheral blood cells) was used to assess assay sensitivity each time the assay was performed. Guthrie card DNA (240 ng) and dilution series of diagnostic DNA from each patient diluted into normal DNA were digested with 5 units *EcoR*V for 16 hours in 10 ul. An additional 5 units of *EcoR*V was added for 2 more hours (18 hours total). The sample was split into six for subsequent PCR analysis (40 ng per reaction). PCR primers D835-BTF (ACATCACAGTAAATAACACTCTGGTG) and D835-BTR (GACACAACACAAAATAGCCGTAT) were used for the SYBR Green PCR backtracking. Nontemplate controls as well as wild-type DNA controls were run alongside patient dilution series and Guthrie DNAs.

For both ITD and point mutations, we utilized SYBR green PCR on an ABI 7900HT with 384 well block: Reactions were performed with 1× buffer (SYBR Green PCR Core Reagents, ABI), 1× AccuPrime PCR buffer, 300 nM of each primer, the appropriate patient-specific forward primer and ITD-R2 (for the ITD reactions) and 3% DMSO, 0.4 U AccuPrime Taq DNA polymerase (Invitrogen), 40 ng template DNA, and water to yield a 10 ul reaction volume. Reactions were performed at 95°C for 5 min, followed by 50 cycles of 94°C for 15 sec, and 60°C for 15 sec. Backtracking PCR reactions were set-up in a different room of the research facility with separately stored PCR reagents from the laboratory where patient DNA was stored and processed for mutation screening, to prevent back-contamination. Test reactions with primers to *ACTB *(beta-actin) were routinely used to confirm the suitability of Guthrie DNA for PCR amplification.

## Results

Eighteen *FLT3 *mutations were discovered among 517 acute leukemia cases for an overall frequency of 3.5%. Nine of 73 were found in AML (12.3% frequency) and 9/441 among ALL patients (2.0%). *FLT3 *mutations were as common in AML-M2 subtype (3 of 20 AML-M2 patients) as in other subtypes (6 of 40 non-AML-M2 patients). Regarding mutation type, insertion/deletion (indel) mutations were more common among AML patients (6 of the 9 indel mutations) than ALL patients (3 of the 9 indels, Table 1). All patients were concurrently assessed for *NRAS *and *KRAS *codon 12 and 13 mutations; one ALL patient had a concurrent *KRAS *mutation with *FLT3 *but no other patients were concurrent for these mutations (Table 1). AML patients with *FLT3 *mutations were older than those without mutations (average 11.5 yrs vs. 7.3 yrs, P = 0.01 by t-test); ALL patients with *FLT3 *were slightly but not significantly older (6.4 vs. 5.5 years average, P = 0.4, t-test). Our observation that indel mutations were more common among AMLs while point mutations were more common among ALLs, suggests a potential complementation or selection of mutation type with cell lineage. Five high hyperdiploid patients had *FLT3 *mutations among 132 in our cases series (3.7%), and four *FLT3 *mutations among 309 non-high hyperdiploid ALLs (1.3%) indicating some bias towards high hyperdiploidy though not significantly (P = 0.13, Fisher's exact test).

**Table 1:** *FLT3 *mutations among 517 acute leukemia subjects from the Northern California Childhood Leukemia Study

Patient ID	ITD or MUT*	MUT	Age	Cytogenetics	FAB (lineage)	Backtrack result^†^
0004	ITD		6.1	46, XY [10/20]; 46, XY, del(9)(p13) [9/20]; 47, XY, +?22 [1/20]	ALL	neg
0087	ITD		10.5	46, XY [21/24]	AML-M1	neg
0104	ITD		9.1	46, XY [19/20]; 44, XY, -14, -22 [1/20]	ALL-L1 (T-cell)	neg
0126	ITD		14.9	46, XX [20/20]	AML-M2	
0201	MUT	GAT→GAA D835E	13.2	46, XY [20/20]	AML-M2	
0261	ITD		13.7	46, XX, t(6;9)(p23;q34) [23/24]	AML-M2	neg
0544 ^‡^	MUT	GAT→GTT D835V	5.3	46, XX [20/20]; nuc ish 12p13(TEL×2), 21q22(AML1×4) [149/207]/12p13(TEL×2), 21q22(AML×2) [31/207]/12p13(TEL×2), 21q22(AML1×3) [25/207]	ALL	neg
0678	ITD		14.5	46, XX [20]	AML-M2	
0738	MUT	TAT→TGT Y842C	5.0	45, XY, -7, del(13)(q13q21) [12/20]; 46, XY [8/20]	ALL-L1	
0745	MUT	GAT→TAT D835Y	12.7	46, XY [21/21]; nuc ish 4cen(CEP4×2), 10cen(CEP10×2), 12p13(TEL×2), 21q22(AML1×2)	ALL-L1/L2	
0796 ^‡^	MUT	GAT→TAT D835Y	1.8	46, XY [3/3]; nuc ish 12p13 (TEL×3), 21q22(AML1×4) [90/100], 12p13(TEL×2), 21q22(AML1×2) [10/100] FISH: +12++21/++X (presumed cryptic high hyperdiploidy)	ALL-L1	neg
0803	MUT	GAT→TAT D835Y and GAT→CAT D835H	0.3	46, XY [20/20]; nuc ish 11q23 (MLL5'x2, MLL3'x2) [200/200]	AML-M5	
0945 ^‡^	MUT	GAT→TAT D835Y	7.9	46, XY [21/21]	ALL	neg
0999	ITD		8.3	46, XX [20]	AML	
1043	MUT	GAT→CAT D835H	14.0	46, XY, inv(16)(p12q22) [12/12]	AML	
1073 ^‡^	DEL		5.9	46, XY [30]; nuc ish 9q34(ABL×2), 22q11.2(BCR×2)	ALL	neg
1107 ^‡§^	MUT	GAT→GCT D835A	3.5	56~58, XY, dup(1)(q21q32),+4,+5,+6,+10,+14,+18,+18,+19,+21, +22,+2mar [5/23]; 46, XY [18/23]	ALL-L1	
1148	ITD		14.5	47, XX, +14 [16]	AML	neg

ITD, internal tandem duplication; MUT, point mutation; DEL, deletion.

**^† ^**neg: 240 ng of patient Guthrie card was tested and was determined to be negative. The rest of the patients were not tested.

^‡ ^Patients exhibiting high hyperdiploidy by FISH assay (see Materials and Methods)

^§^Patient 1107 has a KRAS mutation, which was also negative in backtracking experiment (ref #23)

**Figure 1:**
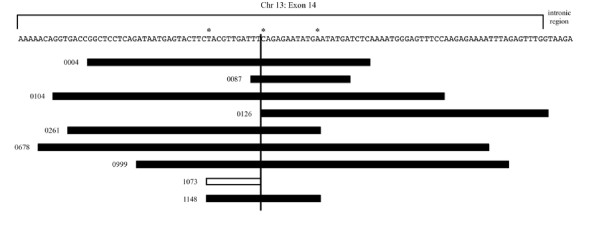
**Relative location and sizes of *FLT3 *ITD mutations and deletion in 9 patients from the Northern California Childhood Leukemia Study**. Black boxes - regions of duplication; white box - deletion of *FLT3 *sequence in one patient. * - positions where multiple breaks occurred.

Eight indel mutations were identified consisting of 8 internal tandem duplications and 1 unconventional deletion. All indel mutants occurred in a 102 base pair region within exon 14 of the *FLT3 *gene, except for one case (0126) whose duplication included one base in the intronic region between exon 14 and 15 (Figure 1 and Additional File 1, Figure S1). Indels ranged from a 9 base pair deletion to a 90 base pair insertion and all preserved the open reading frame. Of the 9 indel mutations found, 6 Guthrie cards were available for backtracking. Each of these 6 Guthrie cards was used with their respective mutant specific primers (Table 2) in an attempt to amplify the DNA and determine if the mutation was present in the birth blood. None of the DNA from the 6 Guthrie cards DNA was amplified using the mutant specific primers at a sensitivity of 1 cell per 6,700 tested in 40 ng DNA (1.5 × 10^-4^) suggesting that the indel mutations were either not present in the DNA from the Guthrie cards, or were present but at a lower frequency than detectable by our assay.

**Table 2:** PCR Primers used for *FLT3 *ITD Backtracking on Guthrie card DNA: Northern California Childhood Leukemia Study

Primer Name	Primer Sequences^‡ ^(5'(3')
ITD-R2	AGACAAATGGTGAGTACGTGCA
ITD-4-F	**ATATGATCTC**tccgagggcc
ITD-87-F	**TATGAATATGA**gTTCAGAGAATATG
ITD-104-F	**GAGTTTCC**cctcgggaag
ITD-261-F	**AGAGAATATGA**GACCGGCTC
ITD-1073-F*	**GAGTACTTC**cc**CAGAGAATATG**
ITD-1148-F	**AGAGAATATGA**gTACGTTGATTTC

^‡ ^**bold**, WT sequence; underlined, ITD; lower case, N nucleotides

*1073 had an 11 bp deletion with a 2 bp insertion, giving an overall deletion of 9 bp.

ITD-R2 was used as the reverse primer for all the backtracking reactions.

Nine point mutations in the *FLT3 *gene were identified with 8 located at codon 835 and 1 located at codon 842. The latter was discovered using Denaturing High Pressure Liquid Chromatography (DHPLC) while the remaining by PCR-RFLP; all were sequenced for confirmation. One patient exhibited two mutations in codon 835 (patient #0803, Table 1). We cloned the PCR product using TA cloning kit (Invitrogen) and sequenced 8 clones - the mutations were present on opposite alleles meaning that two oncogenic mutations existed in this patient. Guthrie cards were available for 4 of these patients, and backtracking experiments were successfully performed on 3 patients at a similar sensitivity as the indel mutations (1 cell in 40 ng DNA), and the three patients were negative for mutant sequence. The requisite reaction sensitivity for one of the patient samples with available cards was not obtained. In sum, between indel and point mutation samples, 9 cards were tested and all were negative for presence of *FLT3 *mutation.

## Discussion

Using NCCLS bone marrow samples, we have screened for *FLT3 *mutations in the largest sample of pediatric leukemias yet reported. Our results confirm the previously reported occurrence of *FLT3 *mutations in both pediatric ALL and AML although the incidence was lower than that of reported for some previous patient series. The highest rates of *FLT3 *mutation were recorded in population series of leukemias in Japan (9% of 162 ALL patients) [28] and Sweden, with 8% of ALL and 21% of AMLs (in children up to age 17 yrs) [9]. Lower frequencies were found in other population series in Greece (2.3% among 86 ALL patients), the UK (3.5% of 86 ALL patients), and Japan (1% of ALL 95 patients) [12]. Our study is the largest *FLT3 *screen in a pediatric population, and was performed in a population-based series of 517 cases. The rate of *FLT3 *among AML patients was 12.3%, which may be lower than some pediatric series since our study includes younger children only (< 15 years). The ALL rate of 2.0% found in here is comparable with the larger more recent reports. It is unlikely that we have missed pathogenetic *FLT3 *mutations in exons 11 and 15 since we incorporated a DHPLC screen of mutations. An additional notable difference between the current study and other studies is the apparent lack of a significant association between high hyperdiploidy and *FLT3 *mutation among our ALL patients, although this lack of significance may be due to small numbers. The association between *RAS *mutations and high hyperdiploidy is extremely strong in our patient sample set [29] and therefore we can confirm a *RAS *pathway association. It is unclear whether a lower prevalence of *FLT3 *mutations among hyperdiploid cases in California (compared to, for instance Paulsson *et al.*, [11]) is due to differences in etiology of this subtype based on genetic or environmental characteristics of patients in California.

Our results suggesting that the *FLT3 *mutation is not present at birth is consistent with the hypothesis that *FLT3 *mutations are a second stage mutation. However, we cannot rule out that the mutations were present in some children but at a level beneath the sensitivity of the assay, or sequestered in the bone marrow and not in blood circulation. Our results corroborate that of Burjanivova, *et al*., who did not find evidence of prenatal origin of *FLT3 *in two AML patients [30]. The "two hit" model of leukemogenesis related to *FLT3 *proposed by Gilliland and Griffin, hypothesizes that two specific types of mutations are required in leukemia: one mutation that promotes proliferation and another mutation that stops differentiation [31]. Mutation of *FLT3*, like other tyrosine kinases is associated with proliferation and may complement mutations that impair cell differentiation such as the deletion of B-cell transcription factors or translocation-associated fusion genes. *NRAS *mutations and *FLT3 *both promote myeloproliferation, so their presence together is not necessary for leukemogenesis. The *MLL *gene rearrangement blocks differentiation and its association with *FLT3 *supports this model as well. None of our *FLT3*-mutant patients exhibited an *MLL*, *TEL-AML1*, *AML1-ETO *or other common translocation, so we were unable to examine a translocation concurrently with *FLT3 *in a backtracking experiment. However, strong prior evidence that high hyperdiploidy is a prenatal event [32-35], combined with no evidence of prenatal origin of *FLT3 *mutation in five high hyperdiploid patients here, helps to place *FLT3 *mutation postnatally.

## Conclusions

In conclusion, *FLT3 *mutations are not common in our California childhood leukemia population, where *RAS *mutations are far more common [29]. Our investigation provided no evidence that *FLT3 *mutation occurs before birth, compatible with a hypothesis of postnatal origin for these mutations.

## Competing interests

The authors declare that they have no competing interests.

## Authors' contributions

All authors have read and approved the final manuscript. PC performed *FLT3 *screening of patients and performed initial backtracking experiments, wrote a first draft of the manuscript, and obtained funding for the study. MK reconfirmed *FLT3 *mutations and performed most of the backtracking experiments. AX assisted in additional point mutation backtracking experiments. JC managed epidemiologic data and performed analysis. JF assisted in the laboratory experiments and interpretation. PB recruited patients and epidemiologic information. JW performed additional backtracking experiments, assisted in the manuscript writing, and also obtained funding.

## Pre-publication history

The pre-publication history for this paper can be accessed here:

http://www.biomedcentral.com/1471-2407/10/513/prepub

## Supplementary Material

Additional file 1 **Figure S1. *FLT3 *ITD Sequences in the Northern California Childhood Leukemia Study**. Exact sequences of internal tandem duplications found in patients from the Northern California Childhood Leukemia Study. Click here for file
