# Supplementary material for: FLT3 mutation incidence and timing of origin in a population case series of pediatric leukemia
Source: BMC Cancer. 2010 Sep 27;10:513. doi: 10.1186/1471-2407-10-513 (PMC2955609; doi:10.1186/1471-2407-10-513)
Supplement: Additional file 1 — Figure S1. FLT3 ITD Sequences in the Northern California Childhood Leukemia Study. Exact sequences of internal tandem duplications found in patients from the Northern California Childhood Leukemia Study. [file 1471-2407-10-513-S1.DOC]

**Additional File 1: Figure S1. *FLT3* ITD Sequences in the Northern California Childhood Leukemia Study.**

Patient 4 (69 bp ITD)

ATGAAAGCCAGCTACAGATGGTACAGGTGACCGGCTCCTCAGATAATGAGTACTTCTACGTTGATTTCAGAGAATATGAATATGATCTC**(tccgaggggccgGGCTCCTCAGATAATGAGTACTTCTACGTTGATTTCAGAGAATATGAATATGATCTC)**AAATGGGAGTTTCCAAGAGAAAATTTAGAGTTTGGTAAGAATGGAATGTGCCAAA

Patient 87 (21 bp ITD)

ATGAAAGCCAGCTACAGATGGTACAGGTGACCGGCTCCTCAGATAATGAGTACTTCTACGTTGATTTCAGAGAATATGAATATGA**(gTTCAGAGAATATGAATATGA)**TCTCAAATGGGAGTTTCCAAGAGAAAATTTAGAGTTTGGTAAGAATGGAATGTGCCAAA

Patient 104 (90 bp ITD)

ATGAAAGCCAGCTACAGATGGTACAGGTGACCGGCTCCTCAGATAATGAGTACTTCTACGTTGATTTCAGAGAATATGAATATGATCTCAAATGGGAGTTTCC**(cctcgggaagggGGTGACCGGCTCCTCAGATAATGAGTACTTCTACGTTGATTTCAGAGAATATGAATATGATCTCAAATGGGAGTTTCC)**AAGAGAAAATTTAGAGTTTGGTAAGAATGGAATGTGCCAAA

Patient 126 (57 bp ITD)

ATGAAAGCCAGCTACAGATGGTACAGGTGACCGGCTCCTCAGATAATGAGTACTTCTACGTTGATTTCAGAGAATATGAATATGATCTCAAATGGGAGTTTCCAAGAGAAAATTTAGAGTTTGG**(CAGAGAATATGAATATGATCTCAAATGGGAGTTTCCAAGAGAAAATTTAGAGTTTGG)**TAAGAATGGAATGTGCCAAA

Patient 261 (51 bp ITD)

ATGAAAGCCAGCTACAGATGGTACAGGTGACCGGCTCCTCAGATAATGAGTACTTCTACGTTGATTTCAGAGAATATGA**(GACCGGCTCCTCAGATAATGAGTACTTCTACGTTGATTTCAGAGAATATGA)**ATATGATCTCAAATGGGAGTTTCCAAGAGAAAATTTAGAGTTTGGTAAGAATGGAATGTGCCAAA

Patient 678 (90 bp ITD)

ATGAAAGCCAGCTACAGATGGTACAGGTGACCGGCTCCTCAGATAATGAGTACTTCTACGTTGATTTCAGAGAATATGAATATGATCTCAAATGGGAGTTTCCAAGAGAAAA**(ACAGGTGACCGGCTCCTCAGATAATGAGTACTTCTACGTTGATTTCAGAGAATATGAATATGATCTCAAATGGGAGTTTCCAAGAGAAAA)**TTTAGAGTTTGGTAAGAATGGAATGTGCCAAA

Patient 999 (81 bp ITD)

ATGAAAGCCAGCTACAGATGGTACAGGTGACCGGCTCCTCAGATAATGAGTACTTCTACGTTGATTTCAGAGAATATGAATATGATCTCAAATGGGAGTTTCCAAGAGAAAATTTA**(cccccccATAATGAGTACTTCTACGTTGATTTCAGAGAATATGAATATGATCTCAAATGGGAGTTTCCAAGAGAAAATTTA)**GAGTTTGGTAAGAATGGAATGTGCCAAA

Patient 1073 (9 bp deletion)

ATGAAAGCCAGCTACAGATGGTACAGGTGACCGGCTCCTCAGATAATGAGTACTTC**(*TACGTTGATTT*cc)**CAGAGAATATGAATATGATCTCAAATGGGAGTTTCCAAGAGAAAATTTAGAGTTTGGTAAGAATGGAATGTGCCAAA

Patient 1148 (24 bp ITD)

ATGAAAGCCAGCTACAGATGGTACAGGTGACCGGCTCCTCAGATAATGAGTACTTCTACGTTGATTTCAGAGAATATGA**(GTACGTTGATTTCAGAGAATATGA)**ATATGATCTCAAATGGGAGTTTCCAAGAGAAAATTTAGAGTTTGGTAAGAATGGAATGTGCCAAA

**(Bold in parentheses)**, complete change in sequence (total ITD); underlined, ITD (duplicated region); lowercase, N nucleotides; *italic*, deleted.
